# Supported Membranes Meet Flat Fluidics: Monitoring Dynamic Cell Adhesion on Pump-Free Microfluidics Chips Functionalized with Supported Membranes Displaying Mannose Domains

**DOI:** 10.3390/ma6020669

**Published:** 2013-02-22

**Authors:** Jochen Oelke, Thomas Kaindl, Andreea Pasc, Zeno Guttenberg, Achim Wixforth, Motomu Tanaka

**Affiliations:** 1 Department of Physics, Technical University Munich, Garching D85748, Germany; E-Mails: joelke@beckman.com (J.O.); kaindl@uni-heidelberg.de (T.K.); andreea.pasc@univ-lorraine.fr (A.P.); 2 Experimental Physics I, University of Augsburg, Augsburg D86159, Germany; 3 Physical Chemistry of Biosystems, University of Heidelberg, Heidelberg D69120, Germany; 4 NANO group-UMR 7565 SRSMC CNRS, Université de Lorraine, Boulevard des Aiguillettes-F54506 Vandoeuvre-Lès-Nancy, France; 5 Beckman Coulter Biomedical GmbH, Advalytix Products, Munich D81377, Germany; E-Mail: herodot@gmx.de; 6 Cell Biophysics Lab, Institute of Toxicology and Genetics, Karlsruhe Institute of Technology, Karlsruhe D76131, Germany

**Keywords:** supported membrane, surface acoustic wave, flatfluidics, cell adhesion

## Abstract

In this paper we demonstrate the combination of supported membranes and so-called flat microfluidics, which enables one to manipulate liquids on flat chip surfaces via “inverse piezoelectric effect”. Here, an alternating external electric field applied to the inter-digital transducers excites a surface acoustic wave on a piezoelectric substrate. Employing lithographic patterning of self-assembled monolayers of alkoxysilanes, we successfully confine a free-standing, hemi-cylindrical channel with the volume of merely 7 µL . The experimentally determined maximum flow velocity scales linearly with the acoustic power, suggesting that our current setup can drive liquids at the speed of up to 7 cm/s (corresponding to a shear rate of 280 s^−1^) without applying high pressures using a fluidic pump. After the establishment of the functionalization of fluidic chip surfaces with supported membranes, we deposited asymmetric supported membranes displaying well-defined mannose domains and monitored the dynamic adhesion of *E. Coli* HB101 expressing mannose-binding receptors. Despite of the further technical optimization required for the quantitative analysis, the obtained results demonstrate that the combination of supported membranes and flat fluidics opens a large potential to investigate dynamic adhesion of cells on biofunctional membrane surfaces with the minimum amount of samples, without any fluidic pump.

## 1. Introduction

Biological membranes are key components of all living systems, forming the outer boundary of living cells or of internal cell compartments (organelles). They consist largely of a lipid bilayer that possesses a fluid character. These features enable membranes to act as smart filter materials. For example, organelles serve as microcontainers to confine biochemical processes inside the cell. Similarly, plasma membranes block most toxic substances from entering into the cell while simultaneously allowing special nutrients, wastes, and metabolites to selectively pass to the outside environment. Moreover, many important biochemical processes occur at membrane surfaces via interactions between various membrane proteins. However, if one looks at biological membranes as a material, they are complex assemblies of a number of molecular machines that we cannot reassemble “piece by piece”.

Since the 1980s, planar lipid membrane models on solid substrates (called supported membranes) are widely used as biological membrane models that can be subjected to various surface sensitive techniques [1,2,3,4]. Membranes deposited on substrates can achieve the coverage of macroscopically large areas (of the order of cm^2^) and the maintenance of excellent mechanical stability, without loosing their fluid nature [3]. Supported membranes can readily be functionalized either by spreading vesicles incorporating transmembrane proteins (proteoliposomes) or by incorporating “anchor” molecules for engineered proteins. This method is a powerful tool for creating complex experimental cell-surface models that can be investigated in a quantitative manner [1,5,6,7,8,9].

An early study of Brian and McConnell *et al.* [1] used model lipid systems to show the recognition of antigen-presenting cells by T cell lymphocytes [10]. Later, Grakoui *et al.* [5] revealed that initiation of the immune response depends on dynamic recognition and interaction during the contact between T cells and the antigen-presenting cell, which is the so-called immunological synapse.

A widely used strategy to monitor the dynamic adhesion of cells under defined shear stresses is to couple substrates functionalized with membranes with a parallel plate microfluidics channel. So far, such systems have been used to monitor the adhesion and rolling of cells on membrane displaying proteins and carbohydrate ligand molecules. However, despite the remarkable success in the past 20 years, commonly used parallel plate fluidics chambers require a large amount of medium and cells for long-term operation, which makes it technically difficult to study the adhesion behaviors under various conditions.

The concept of “lab-on-a-chip” is one straightforward strategy to perform such fluidics experiments with miniaturized devices by means of the etching of channels deep into glass or the mounting of polymer-based top covers [11,12,13]. However, such strategies encounter the fundamental problem that an enormous pressure must be applied to push liquid through the micro-channels. To avoid the problems of micro-channel systems, we developed a microfluidic system on a planar chip made of piezoelectric crystal that is operated by surface acoustic wave (SAW) actuation [14]. The manipulation of liquids on a chip relies on the “inverse piezoelectric effect”, where an alternating external electric field at the transducer electrodes on a piezoelectric substrate excites a periodic mechanical wave. Due to viscous damping, the conversion of SAW within the fluid bulk into a longitudinal sound wave and the generation of an acoustic radiation pressure cause acoustic streaming. This technology, often called “flat micro-fluidics”, has been used widely to transport/mix fluids in various fields, such as array dispensers for DNA hybridization [15], by utilizing the hydrophobic/hydrophilic contrasts on the chip surfaces. Flat fluidics has also been coupled to top covers made of poly(dimethylsiloxane) (PDMS) and used for the adhesion of proteins [16], vesicles, biopolymers [17] and cells. The recent developments in the field of acoustically induced microfluidics are nicely comprised in a recent review article by Friend *et al.* [18].

In this paper, we report the functionalization of a flat fluidics device with an asymmetric supported membrane displaying functional micro-domains and monitor the dynamic adhesion of bacteria. In contrast to previous studies relying on PDMS covers, a free-standing, hemi-cylindrical liquid channel was lithographically fabricated by generating the hydrophobic/hydrophilic contrast aligned in a circular layout to form a closed channel. This enables one not only to apply liquid at controlled shear rates by acoustic streaming without connecting the liquid channel to a tubing or pump but also to reduce the total liquid volume to as little as 7 µL. We first calibrated the relationship between shear rate and streaming velocity using latex particles. In the next step, we deposited asymmetric supported membranes displaying mannose micro-domains and monitored dynamic adhesion of bacteria expressing mannose binding receptors.

## 2. Materials and Methods

### 2.1. Materials

1,2-Dioleoyl-*sn*-glycero-3-phosphocholine (DOPC) was purchased from Avanti Polar Lipids Inc. (Alabaster, AL, USA), and 1,2-dihexa-decanoyl-*sn*-glycero-3-phosphoethanolamine, triethylammonium salt (TexasRed-DHPE) from Invitrogen (Karlsruhe, Germany). The synthesis of fluorinated lipid molecules followed the previously reported synthetic pathway [19,20,21], and details of synthesis are described in our previous account [22].

**Figure 1:**
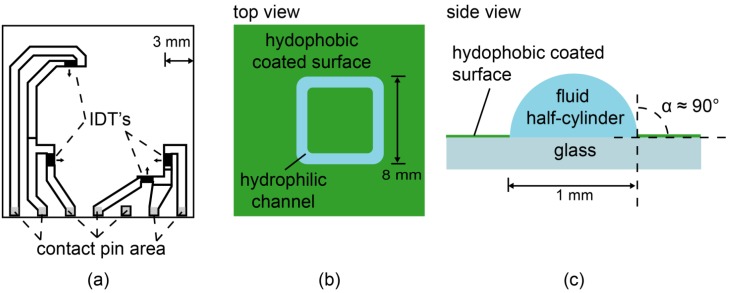
(**a**) Sketch of the inter-digital transducer (IDT) layout deposited on bare LiNbO_3_ 128° Rot *Y*-Cut. The small arrows indicate the direction of the surface acoustic wave (SAW) propagation. The surface is covered with SiO_2_ for further chemistry. The top view (**b**) and the side view (**c**) of a hydrophilic channel (blue) surrounded by the surface coated with hydrophobic silanes.

All the fluidic chips used in this study were manufactured by Advalytix (Munich, Germany). The chip is based on LiNbO_3_ crystal that is cut in an angle of 128° in respect to the *Y* axis. It exhibits a strong SAW propagation direction along the crystal *X* axis and the best electromechanical coupling coefficient for Rayleigh waves [23]. The bare crystal was structured with a circuit layout of inter-digital transducers (IDTs, Figure 1a) to launch SAW in the four perpendicular directions in the *x*–*y* plane. The chip surface was coated with SiO_2_ to allow for further surface chemistry.

### 2.2. Surface Chemistry, Lithographic Structuring of Fluidic Channels

The chips were cleaned by using a modified RCA (Radio Corporation of America) protocol [24]: The samples were sonicated for 5 min in acetone, ethanol, methanol, and water, then immersed in a solution of H_2_O_2_ (30%)/NH_4_OH (30%)/H_2_O (1:1:5 by volume) and sonicated for 5 min at room temperature before being soaked for another 30 min at 60 °C. Afterwards, they were intensively rinsed with water, dried at 70 °C, and stored in a vacuum chamber. Chip surfaces were first hydrophobized with octadecyltrimethoxysilane (ABCR GmbH, Karlsruhe, Germany) as reported previously [25], and spin-coated with Shipley 1813 positive photoresist (5000 rpm, 30 s). After the removal of solvent, the structure mask was applied by a MJB3 mask aligner (Süss MicroTec AG, Garching, Germany) to irradiate the channel for 30 s. In the next step, photoresist from the irradiated region was removed, and the silane monolayers were removed by oxygen plasma (200 mbar, 40 s). Finally, the residual photoresist was removed by sonication in acetone for 3 min, resulting in a free-standing, hemi-cylindrical fluidic channel (Figure 1b,c).

## 3. Results and Discussion

### 3.1. Modeling of Flow Profiles

**Figure 2:**
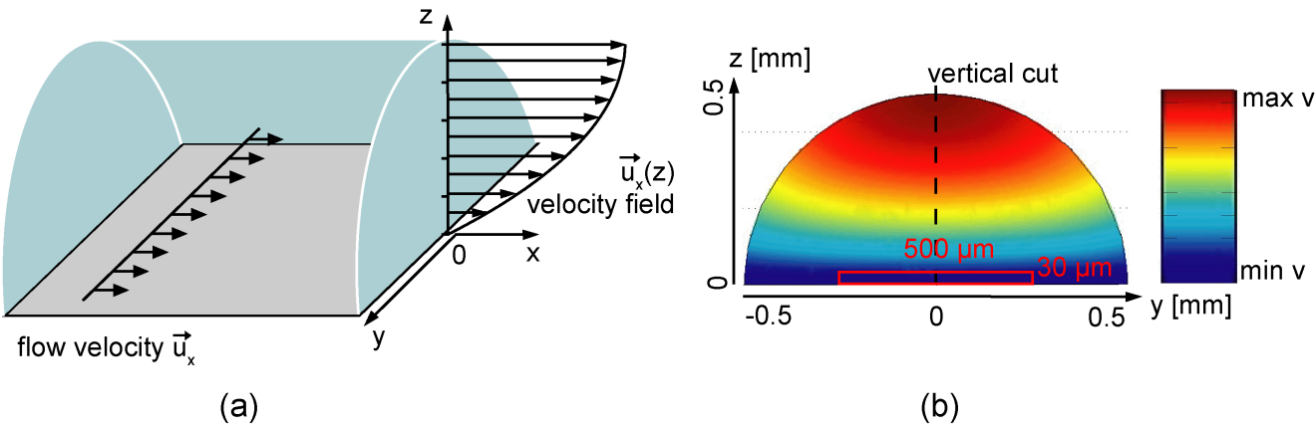
(**a**) Scheme of the flow geometry; (**b**) the cross-sectional view of the calculated flow velocity profile.

To calculate the flow profile in the hemi-cylindrical channel geometry as being presented in Figure 2a, we implemented the finite elements modeling tool FEMLAB (COMSOL Multiphysics) by considering the following boundary conditions: (1) no-slip boundary condition at the solid-liquid interface, *i.e.*, *u*(*z* = 0) = 0; and (2) slip boundary condition at the air/liquid interface, *i.e.*, *u*(*z* = *h*, *y* = 0) = *u*_max_. Figure 2b represents the cross-sectional view of the calculated velocity field calculated by FEMLAB. The flow velocity increases according to the increase in *z* (the distance from the substrate), but the velocity near the center remains almost constant along *y* axis. Therefore, we selected the region of interest (indicated as a box) near the center and close to the surface.

**Figure 3:**
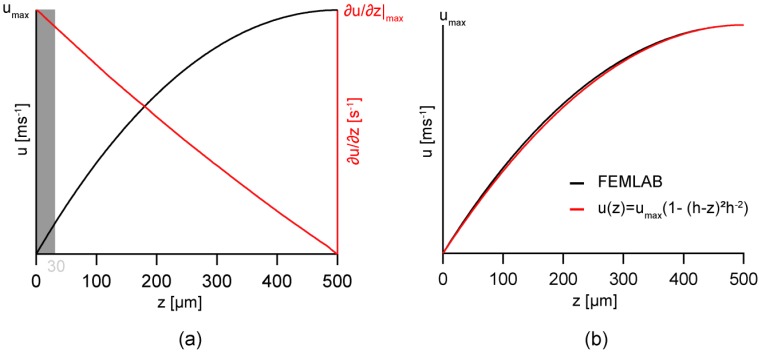
(**a**) The flow velocity *u* (black line) and its first derivative ∂*u*/∂*z* (red line) in the middle of the channel (*y* = 0), calculated by FEMLAB; (**b**) The flow velocity profile (black line) fitted with the Hagen-Poiseuille flow profile (red line).

Figure 3a represents the flow velocity *u* and its first derivative ∂*u*/∂*z* with respect to *z* in the middle of the channel, *y* = 0, calculated by FEMLAB. In case of a infinitely extended channel in *x*-direction with fixed pressure gradient, the velocity profile at *y* = 0 can be well fitted by a parabolic function Figure 3a, left axis), called Hagen-Poiseuille flow profile [26]:

$$u(z)=u_{\text{max}}[1-{\left(\frac{\left(h-z\right)}{h}\right)}^{2}]$$

where *u*_max_ is the maximum velocity and *h* the height of the channel. In fact, the first derivative of the velocity with respect to *z* calculated by FEMLAB (Figure 3a, right axis) seems to scale linearly with *z*.

Following Newton’s second law, the shear rate *γ* is proportional to the first derivative of the flow velocity

$$\gamma =\frac{du}{dz}$$

As presented in the figure, the maximum share rate *γ*_max_ is achieved at the bottom of the channel, *i.e.*, the solid/liquid interface. The region of interest is indicated by the shaded zone in Figure 3a corresponding to the height of *z* = 0–30 µm. Within the selected range, the deviation in the maximum share rate along the *y* axis is approximately 5%. This enables one to calculate the shear rate from the maximum velocity measured at the top of the hemi-cylindrical channel.

The choice of the pressure-driven flow model presented in this study has been validated by our previous theoretical models [27,28]. In these accounts, we have shown that the assumption of a homogeneous, though locally confined body force field leads to a very good description of our experimental findings based on acoustically driven streaming.

### 3.2. Calibration of Shear Rates by Acoustic Streaming

Prior to the experiments with cells on functionalized fluidic chips, the shear rate exerted in the hemicylindrical channel is calibrated by measuring the translational velocity of fluorescently labeled latex particles with a diameter of 1 µm (Sigma-Aldrich) on a bare chip surface. The acoustic streaming is performed at a frequency of 100–200 MHz through a frequency generator (Advalytix AG) controlled by a Labview interface. Here, we set the maximum acoustic level *A*_max_ at 20 dBm (*P*_max_ = 100 mW) [29], made 256 discrete steps (including zero), and used these step numbers *N* as preliminary indicators for the acoustic level *A*: *A* = 10 × log(*P*_max_ × (*N*/255)/1 mW). In the next step, the displacement of the tracer beads near the top of the channel is monitored by a Firewire CCD Camera connected to an upright microscope (Helmut Hund GmbH, Wetzlar) equipped with a 20× long distance objective (Carl-Zeiss, Oberkochen) at a frame rate of 30 fps, and the velocity *u* of each bead is tracked manually.

**Figure 4:**
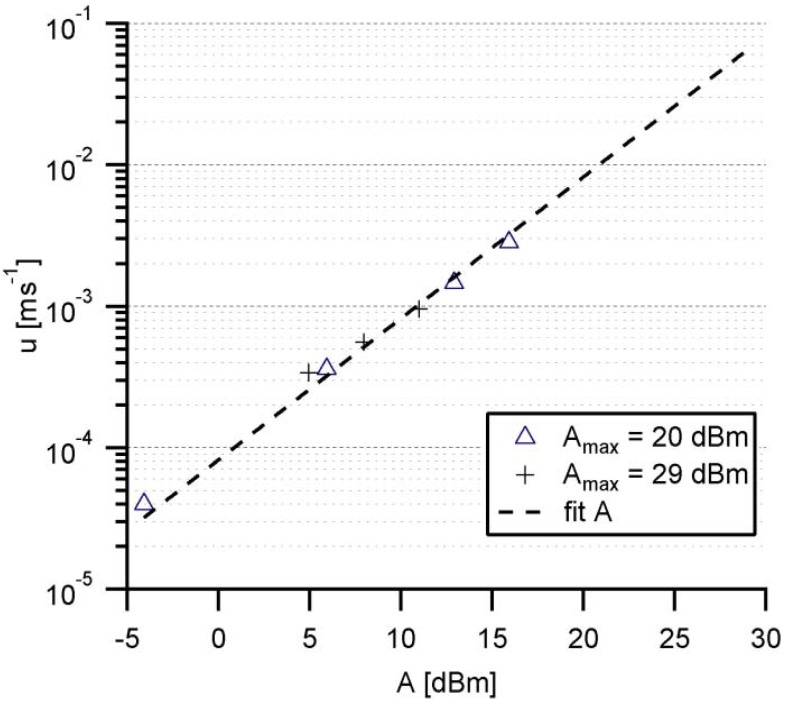
Experimentally determined streaming velocity *u* plotted on log-scale as the function of acoustic level *A*, obtained from two sets of experiments at *A*_max_ = 20 dBm and 29 dBm, respectively.

In this series of experiments, we tracked the displacement of the beads at *N* = 1 (minimal), 10, 50, and 100 steps. Figure 4 represents the experimentally determined velocity *u* as the function of *A*, verifying the logarithmic relationship between *u* and *A*. The linear extrapolation of the plot to *A*_max_ = 20 dBm demonstrates that our pump-free flat fluidic setup can drive the fluid at the velocity of up to 0.8 cm/s. In fact, the limit of the current setup, *i.e.*, 29 dBm (*P*_max_ = 800 mW), allows fluid velocities up to 7 cm/s, although the corresponding shear rates are beyond physiological relevant values for cell adheasion experiments. The maximal *A*_max_ settings coincide with a maximum shear rate of *γ*_max(20 dBm)_ = 32 s^−1^ and *γ*_max(29 dBm)_ = 280 s^−1^, respectively, and result in a theoretical shear stress τ for adherent cells at 37 °C up to τ_(20 dBm)_ = 0.22 dyn cm^−2^ and τ_(29 dBm)_ = 1.93 dyn cm^−2^.

### 3.3. Position Selective Functionalization of Fluidic Chips with Supported Membranes

**Figure 5:**
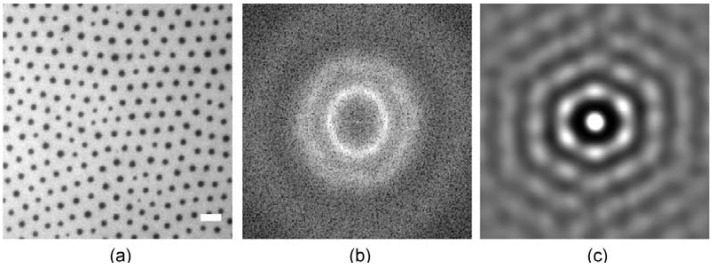

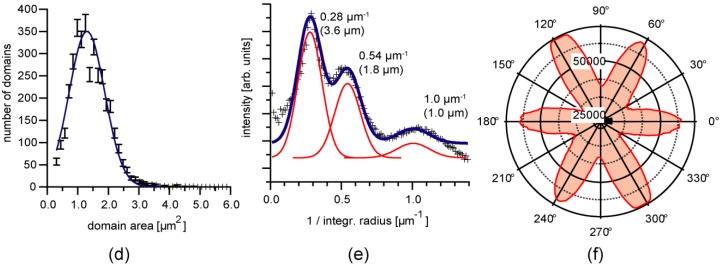
Structural characterization of FL10man domains (33 mol %) incorporated in a 1,2-Dioleoyl-*sn*-glycero-3-phosphocholine (DOPC) monolayer; (**a**) fluorescence image; (**b**) spectral density plot; (**c**) autocorrelation; (**d**) domain size distribution; (**e**) radial integration; and (**f**) polar intensity plot of nearest neighbors.

In the first step of membrane preparation, the FL10man domains are characterized at the air/water interface. Figure 5a shows the fluorescence image of FL10man domains (33 mol %) incorporated in a DOPC monolayer. The fluorescence image analysis enables one to gain the domain size distribution (Figure 5d) and thus the mean area and radius of FL10man domains, *A*_FL10man_ = 1.30 ± 0.02 mm^2^ and *R*_FL10man_ = 0.64 ± 0.05 µm, respectively. The spectral density plot (Figure 5b) and its radial integration (Figure 5e) can be fitted with multiple Gaussian functions, yielding the inter-domain distance *a* = 3.60 ± 0.05 µm, and two characteristic lengths scales *L*_1_ = 1.80 ± 0.03 µm, and *L*_2_ = 1.00 ± 0.10 µm. The sharp spatial confinement of domains is well visible from the autocorrelation (Figure 5c) and the polar intensity plot of nearest neighbors (Figure 5f) that exhibits clear positive peaks.

**Figure 6:**
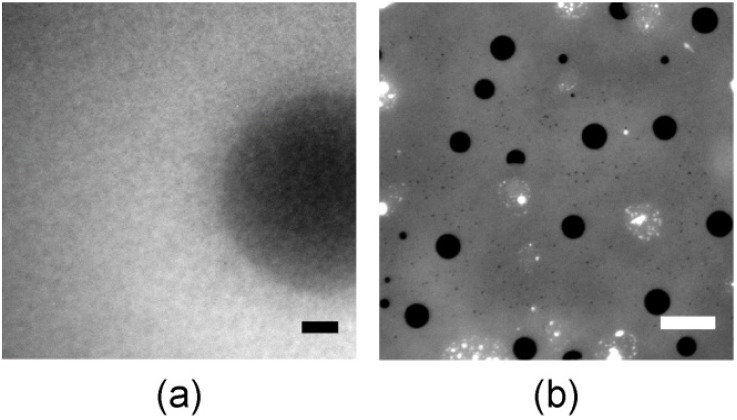
(**a**) Fluorescence image of a supported membrane prepared by the fusion of DOPC vesicles (diameter ~100 nm) incorporating 33 mol % FL10man and (**b**) after solvent exchange. Scale bars are 10 µm.

A commonly used method to deposit supported lipid membranes is the fusion of vesicles directly on solid substrates. Figure 6a represents the fluorescence image of a supported membrane prepared by the fusion of DOPC vesicles (diameter ~100 nm) incorporating 33 mol % FL10man. As presented in the figure, no distinct domain can be found, suggesting that the perfluorinated FL10man form own aggregates due to the lipophobicity of perfluorinated lipids [30,31]. Another possible scenario is that small vesicles are not mechanically stable if they incorporate domains larger than the vesicle size. Another preparation method using the stepwise exchange of polar solvents only resulted in disrupted membranes where domains could not be controlled in size and inter-domain distance (Figure 6b).

**Figure 7:**
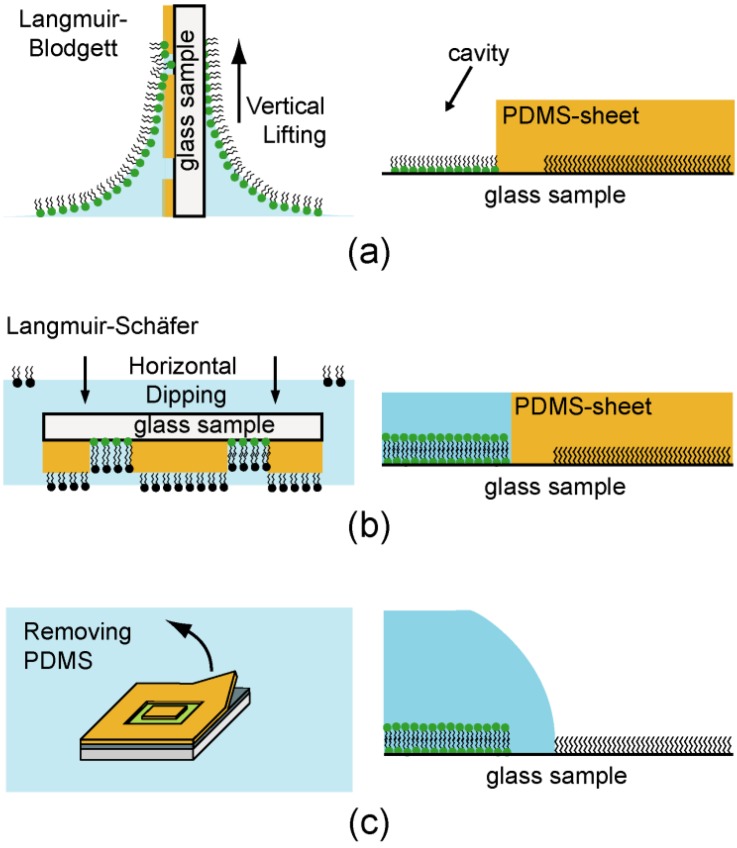
The stepwise functionalization of a µ-fluidic chip with asymmetric supported membranes. To sustain stable hemi-cylindrical channels, the membrane patches and the surrounding silane region were separated via bare SiO_2_ surface (width ~200 µm).

To avoid any undesired exclusion of functional domains, an asymmetric supported membrane was prepared by the successive deposition of the proximal and distal monolayer. Although this method is much more laborious than a fusion of small unilamellar vesicles, this enables one to fabricate lipid bilayers with an asymmetric lipid compositions [32]. For the deposition of the proximal monolayer, a substrate partially covered with a PDMS protection layer was immersed into the subphase, and the lipid stock solution was spread onto a water subphase of a Langmuir film balance (Nima Technology Ltd., Coventry, UK). After evaporation of the solvents, the film was compressed at a low speed (0.01 Å^2^ molecule^−1^ s^−1^) to a surface pressure of π = 20 mN m^−1^ at *T* = 293 K. The proximal layer was transferred by vertically pulling the substrate (Figure 7a) from the water subphase at a constant surface pressure (Langmuir-Blodgett transfer). After the transfer onto a hydrophobic glass slide by horizontal dipping (Figure 7b) into the subphase (Langmuir-Schaefer transfer), the PDMS protection sheet was removed (Figure 7c), resulting in a supported membrane confined in a free-standing, hemi-cylindrical fluidic channel. We confirmed the formation of homogeneous, fluid lipid membranes from the lateral mobility of matrix lipids by the fluorescence recovery after photobleaching (FRAP), as reported previously [22]. It should be noted that the membrane should be in contact with water after the second transfer. To avoid the membrane disruption by evaporation of water, the chip was always kept in a sealed container.

### 3.4. Cell Adhesion under Various Shear Conditions

**Figure 8:**
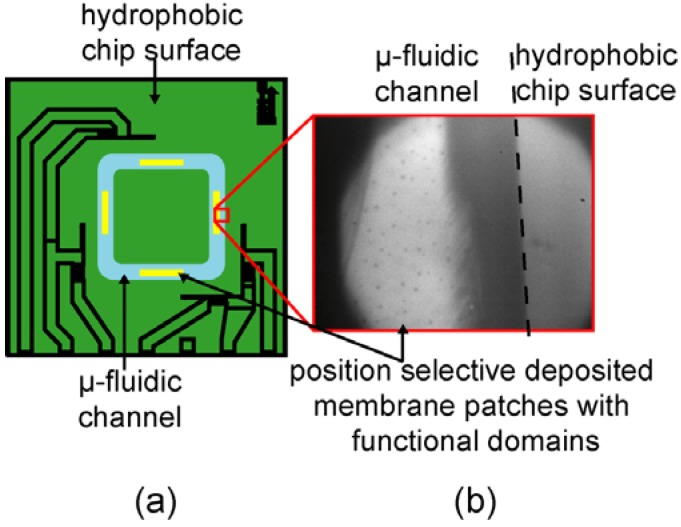
(**a**) Schematic illustration of the µ-fluidic chip layout. The regions highlighted in yellow are functionalized with asymmetric supported membranes; (**b**) fluorescence image of the supported membrane displaying domains of FL10man (black dots).

The adhesion of *E. Coli* HB101 (pPKl4) expressing a mannose-binding receptor was studied under various shear conditions. For the preliminary tests, two types of supported membranes were subjected to the adhesion experiments: The specific adhesion was studied using supported membranes displaying micro-domains of mannose [22]. The proximal leaflet of the membrane contains 5 mol % FL10man that results in the average domain radius of 0.58 ± 0.1 µm and the inter-domain distance of 10.3 ± 1.8 µm. The control experiment was performed with pure DOPC membranes. Figure 8 represents (a) an overview of the chip layout and (b) a zoom-up image of the self-assembled FL10man domains embedded in the patches of membranes deposited near the middle of the channel.

**Figure 9:**
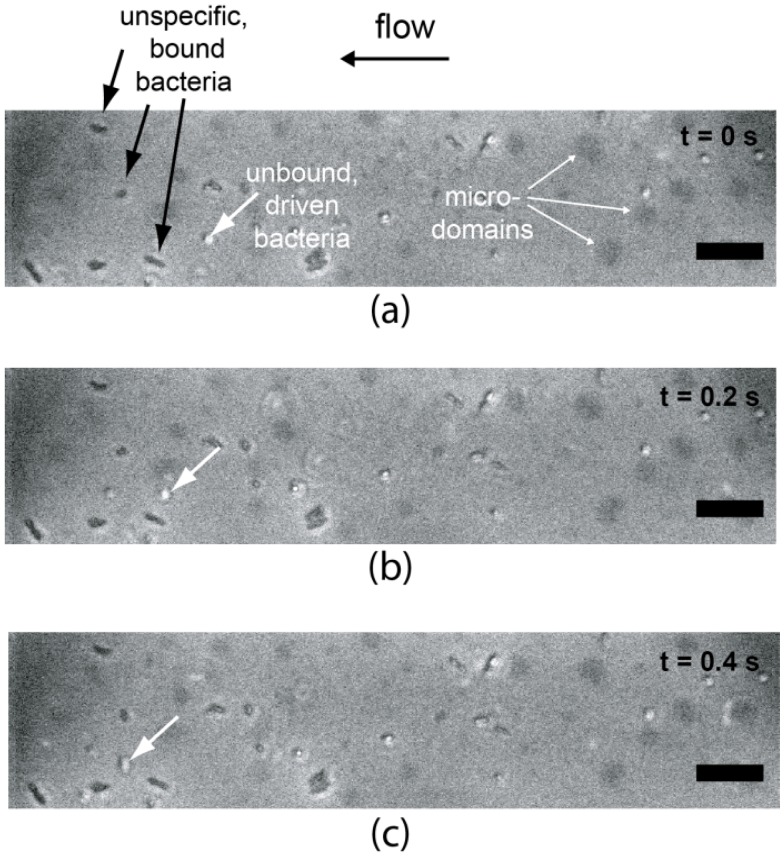
Overlay of phase contrast and fluorescence images of *E. Coli* HB101 on a supported membrane displaying FL10man domains (black patches) under a shear rate of 2.7 s^−1^. The scale bars are 25 µm.

Figure 9 shows the overlays of phase contrast and fluorescence images of *E. Coli* HB101 on a supported membrane displaying FL10man domains under a shear rate of 2.7 s^−1^. Here, the fluorescence images are used to visualize FL10man domains (black patches) and the phase contrast images to monitor the displacement of *E Coli.* As indicated by arrows in the figure, we could observe the coexistence of cells bound to the membrane surfaces (white arrows) and cells that are not bound and thus flow inside the fluidic channel. In contrast on pure phospholipid (DOPC) membranes, we observed no sign of specific adhesion.

The experimental findings clearly indicate that our cover-free flat fluidic chips coated with asymmetrically functionalized supported membranes are feasible to monitor dynamic cell adhesion in an extremely small liquid volume (7 µL) under defined shear rates without any pump. Unfortunately, the current image resolution of our setup does not enable us to conclude whether *E. Coli* adheres specifically onto FL10man domains. To overcome this problem, we are in the process of implementing dark field illumination to our fluidic workstation to gain sharper images of cells and to study the impact of domain size and distribution by changing the concentration of lipid anchors and chemically modifying fluorinated lipid anchors.

## 4. Conclusions

The combination of supported membranes and flat microfluidics is a promising strategy to study dynamic cell adhesion processes by pushing liquids on flat chip surfaces via “inverse piezoelectric effect” without applying high pressures using a fluidic pump. In this paper, we described how one can confine a cover-free, hemi-cylindrical channel with the volume of merely 7 µL with aid of lithographic patterning and surface chemistry. A linear relationship between maximum flow velocity and acoustic power suggests that our current setup can drive liquids at the speed of up to 7 cm/s (corresponding to a shear rate of 280 s^−1^). As a preliminary attempt to study dynamic cell adhesion on micro-patterned supported membranes on flat fluidics chips, we deposited asymmetric supported membranes displaying highly uniform mannose domains. Although there is a large room of further technical improvements, we could monitor the dynamic adhesion of *E. Coli* HB101 expressing mannose-binding receptors. The obtained results demonstrate that the new strategy proposed in this paper would allo0w for the “pump-free” investigation of dynamic adhesion of cells on biofunctional membrane surfaces with the minimum amount of samples.
